# Delamination from an adhesive sphere: Curvature-induced dewetting versus buckling

**DOI:** 10.1073/pnas.2212290120

**Published:** 2023-03-17

**Authors:** Finn Box, Lucie Domino, Tiago Outerelo Corvo, Mokhtar Adda-Bedia, Vincent Démery, Dominic Vella, Benny Davidovitch

**Affiliations:** ^a^Mathematical Institute, University of Oxford, Oxford OX2 6GG, UK; ^b^Department of Physics and Astronomy, University of Manchester, Manchester M13 9PL, UK; ^c^Institute of Physics, Universiteit van Amsterdam, Amsterdam 1098 XH, The Netherlands; ^d^Laboratoire de Physique, Univ Lyon, École Normale Supérieure de Lyon, CNRS, Lyon 69342, France; ^e^Gulliver, CNRS, École Supérieure de Physique et Chimie Industrielles Paris Paris Science et Lettres, Paris 75005, France; ^f^Department of Physics, University of Massachusetts, Amherst, MA 01003

**Keywords:** geometric incompatibility, adhesion, thin sheets

## Abstract

Everyday experience confirms the tendency of adhesive films to detach from spheroidal regions of rigid substrates—what is a petty frustration when placing a sticky band aid onto a knee is a more serious matter in the coating and painting industries. Irrespective of their resistance to bending, a key driver of such phenomena is Gauss’ *Theorema Egregium*, which implies that naturally flat sheets cannot conform to doubly curved surfaces without developing a strain whose magnitude grows sharply with the curved area. Previous attempts to characterize the onset of curvature-induced delamination, and the complex patterns it gives rise to, assumed a dewetting-like mechanism in which the propensity of two materials to form contact through interfacial energy is modified by an elastic energy penalty. We show that this approach may characterize moderately bendable sheets but fails qualitatively to describe the curvature-induced delamination of ultrathin films, whose mechanics is governed by their propensity to buckle and delaminate partially, under minute levels of compression. Combining mechanical and geometrical considerations, we introduce a minimal model for curvature-induced delamination accounting for the two buckling motifs that underlie partial delamination: shallow “rucks” and localized “folds”. We predict nontrivial scaling rules for the onset of curvature-induced delamination and various features of the emerging patterns, which compare well with experiments. Beyond gaining control on the use of ultrathin adhesives in cutting-edge technologies such as stretchable electronics, our analysis is a significant step toward quantifying the multiscale morphology that emerges upon imposing geometrical and mechanical constraints on highly bendable solid objects.

## Significance

Adhering two materials with different intrinsic curvatures is desirable in settings from scientific measurements to the development of epidermal electronics, as well as in everyday procedures (like applying a band-aid to a child’s knee). However, the geometry of curved surfaces and the mechanics of thin materials make this, apparently simple, procedure difficult: Adhering two materials of different Gaussian curvature requires incompatible deformations. Nevertheless, sufficiently small sheets smoothly adhere to, for example, a sphere. We study in detail the threshold size below which smooth adhesion is possible and show that for very thin materials its failure occurs significantly sooner than expected from previous theoretical work. This earlier onset is the signature of buckling-induced delamination and not the dewetting-like transition previously assumed.

The simplest way to form a composite material, simply sticking a layer of one material to another, is encountered in everyday life from sticky notes to a parent placing a band-aid on a child’s knee. Normally one does not consider whether such an operation is at all possible. However, when at least one of the two objects to be joined is curved, adhesion is no longer guaranteed. For example, when a relatively thick, flat plate is adhered to a cylindrical substrate, the energetic penalty associated with detaching from the substrate is small enough to be overcome by the elastic (bending) energy of the plate that is released by detachment (1) and leads to the failure of the coating or delamination. For sufficiently thin plates, this bending energy is insignificant and adhesion proceeds as expected.

**Fig. 1.**
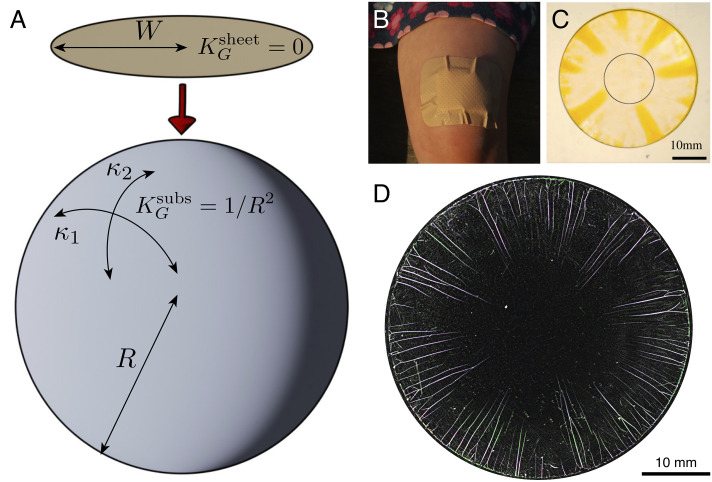
Illustrations of the geometric incompatibility of an intrinsically flat sheet adhering to a doubly curved object. (*A*) A circular, naturally flat, sheet of radius *W* has Gaussian curvature *K*_*G*_^sheet^ = 0 while a spherical substrate of radius *R* has Gaussian curvature *K*_*G*_^subs^ = *κ*_1_*κ*_2_ = 1/*R*^2^. (*B*) Delamination blisters spontaneously form when a (flat) band-aid is adhered to a child’s (curved) knee. Panels *C* and *D* show more controlled realizations of this experiment in sheets of large and small thicknesses, respectively. Image in C reprinted with permission from (3). Copyright (2011) by the American Physical Society.

A qualitatively different picture emerges when the substrate is doubly curved (i.e., has two principal curvatures *κ*_1, 2_ ≠ 0, Fig. 1*A*). While very thin sheets are able to bend easily, stretching is much more difficult and so Gauss’ *Theorema Egregium* (2) limits them to maintaining their initial Gaussian curvature, *K*_*G*_^sheet^. If the Gaussian curvature of the substrate *K*_*G*_^subs^ = *κ*_1_*κ*_2_ ≠ *K*_*G*_^sheet^, adhesion between the two frustrates the deformable object (sheet). If the sheet nevertheless adheres perfectly to the surface, it suffers a strain that scales with the contact area and hence an elastic energy density that is quadratic in the sheet’s area. Consequently, it has been commonly assumed that a thin sheet delaminates from a spheroidal-shaped substrate when its size reaches a threshold value at which the penalty of this strain-induced elastic energy exceeds the gain in adhesion energy. The central result of our paper is that, while this “ideal delamination” scenario (which we liken to dewetting) may characterize moderately bendable sheets, it does not apply for highly bendable sheets. These are governed instead by “partial delamination” in which localized “rucks” and “folds” appear. Crucially, partial delamination occurs for sheets that are much smaller than is suggested by the hypothetical energy-based threshold and largely suppresses the elastic energy penalty, thereby considerably increasing the net coverage of spheroidal parts of the substrate.

The geometrical frustration resulting from a difference in Gaussian curvature occurs when a flat sheet is deposited on a sphere, as is shown schematically in Fig. 1*A* and in a practical scenario in Fig. 1*B*. For the example shown in Fig. 1*B*, this incompatibility leads to failure in the form of delamination blisters forming around the periphery. While this is a minor annoyance in the example of a band-aid applied to a curved knee, in technological and scientific applications conformability is key and so delamination is problematic (4–7). As a result, a variety of techniques have been developed to overcome geometric incompatibility ranging from modifying the substrate geometry as in the Surface Force Apparatus (8, 9) to buffering the excess area required to change the sheet’s shape (10) by either removing material (7, 11, 12) or introducing sacrificial buckling elements (13, 14).

Despite the broad significance of geometric incompatibility for adhesion, there seems to be little detailed understanding of when and how this incompatibility is expected to lead to failure via delamination. The standard picture of delamination induced by geometric incompatibility is due to Majidi and Fearing (15), who observed that if a flat sheet (of radius *W*) is forced to adhere to a sphere of radius *R*, a strain of order *ε* ∼ *K*_*G*_^subs^*W*^2^ ∼ *W*^2^/*R*^2^ is induced. Perfect adhesion therefore induces an elastic energy density *Y*(*W*/*R*)^4^, where *Y* = *Et* (with *E* the Young modulus and *t* the sheet thickness) is the stretching modulus of the sheet. Now, this elastic energy penalty can be avoided if the sheet retains a planar shape by completely detaching from the substrate, at the expense of paying an energetic penalty *Γ* per unit area of lost contact.

Assuming the sheet is either fully adhered to the substrate or fully delaminated from it, one may define a renormalized, curvature-dependent adhesion energy density:

[1] $$\begin{array}{c} \Gamma^{*}=\Gamma -c\cdot Y{\left(W/R\right)}^{4}, \end{array}$$

where *c* is a numerical constant that depends on the substrate geometry; for example, *c* = 1/384 for a spherical substrate (16, 17). Eq. **1** underlies an elementary description of delamination as a generalization of the standard dewetting transition (which occurs as *Γ* → 0^+^), to “curvature-induced dewetting” (which occurs as Γ^*^ → 0^+^). In this generalization, the sheet’s elastic energy is simply viewed as an addition to the sheet–substrate adhesion energy. In terms of the dimensionless parameters:

[2] $$\begin{array}{c} \tilde{W}=W/R\;\;;\;\;\beta =\Gamma /Y, \end{array}$$

the curvature-induced dewetting scenario predicts that delamination occurs when $\tilde{W}$ exceeds a critical value:

[3] $$\begin{array}{c} {\tilde{W}}_{\text{dewet}}∼\beta^{1/4}. \end{array}$$

Although the curvature-induced dewetting mechanism does capture the basic competition between adhesion and geometrical constraints, considering Γ^*^, defined in Eq. **1**, as the single parameter that determines the onset of delamination is problematic. Indeed, treating adhesion and elastic energies as equivalent competitors obscures the fact that the former is uniformly distributed while the latter is distributed in a nontrivial and inhomogeneous manner: Not only does the magnitude of strain vary significantly with radial distance from the center, but the spatial structure of its components is quite nontrivial. In particular, while the inner part of the sheet is stretched both radially and azimuthally, the periphery is stretched predominantly radially and becomes azimuthally compressed when the sheet exceeds a critical size (16, 17):

[4] $$\begin{array}{c} {\tilde{W}}_{\text{comp}}∼\beta^{1/2}. \end{array}$$

Notably, for *β* ≪ 1, ${\tilde{W}}_{\text{comp}}$ may be significantly smaller than the delamination size ${\tilde{W}}_{\text{dewet}}$ predicted by the curvature-induced dewetting mechanism, Eq. **3**. Since the thinner a sheet is the less compression it can accommodate before buckling, one may expect that the onset of curvature-induced delamination in sufficiently thin sheets is not correctly described by the single parameter Γ^*^ but requires an explicit consideration of the strong nonuniformity and anisotropy, and the consequent possibility of anisotropic instabilities, similar to the radial wrinkling that is prevalent in other examples of geometrically incompatible confinement problems (10, 18, 19). The possible relevance of such a strongly anisotropic response to delamination is clear in Fig. 1 *C* and *D*, which shows that delamination occurs via the formation of radial blisters.

**Fig. 2.**
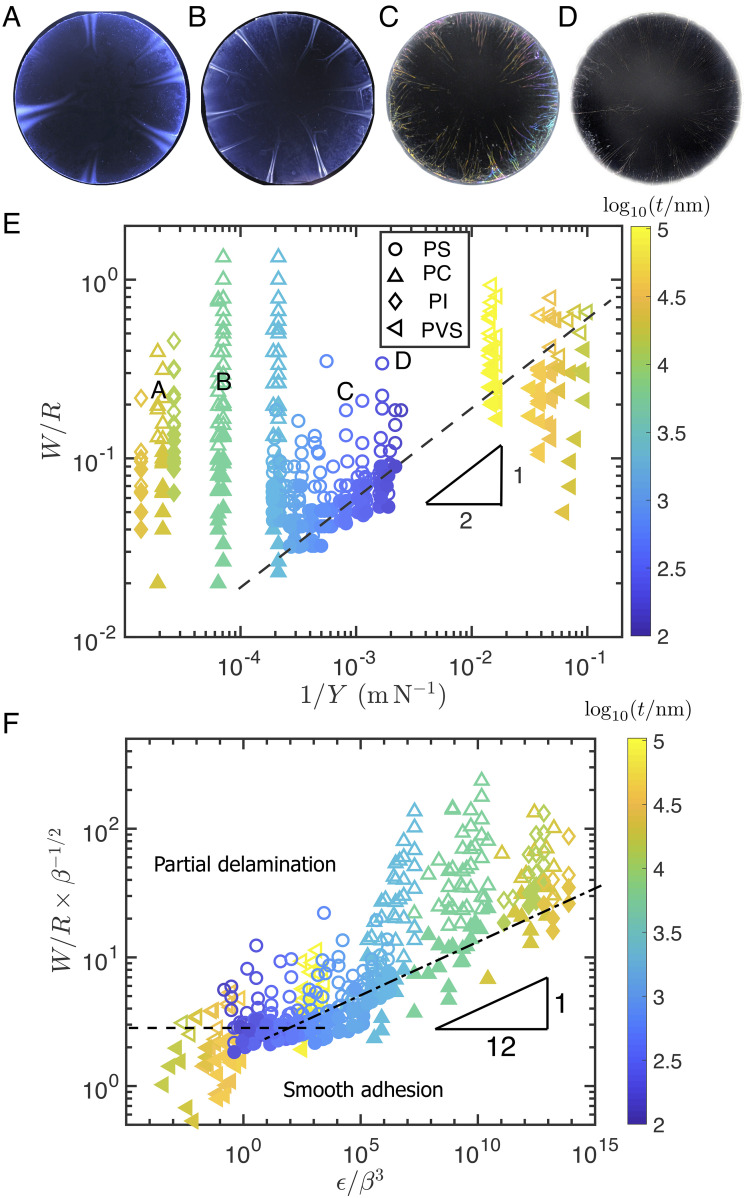
*A*–*D*: Images showing typical delamination patterns for a range of sphere sizes and sheet thicknesses (as summarized by the letters *A*–*D* in panel *E*). (*E*) Initial presentation of experimental results showing the regions of $\left(1/Y,W/R=\tilde{W}\right)$ parameter space for which smooth adhesion (filled symbols) or localized delamination (open symbols) were observed. Contrary to previous suggestions, the delamination transition does not occur when ${\tilde{W}}_{c}=384^{1/4}\beta^{1/4}\propto {\left(1/Y\right)}^{1/4}$. Instead, the theory presented in this paper suggests different regimes depending on the value of *ϵ*/*β*^3^. (*F*) Replotting the data of panel *E* as suggested by this theory shows a good collapse of the data as well as the two distinct regimes predicted theoretically. The scaling prediction for *ϵ*/*β*^3^ ≪ 1, Eq. **15**, is shown by the dashed line and is observed even with moderate *ϵ*/*β*^3^ ≲ 10^2^; the prediction for *ϵ*/*β*^3^ ≫ 1, Eq. **23**, is shown by the dash-dotted line. In panels *E* and *F*, points are shown for a variety of sheet thicknesses, indicated by point color (see colorbar to the right), and material, indicated by shape: polyimide (diamond), polycarbonate (upward-pointing triangles), polyvinylsiloxane (left-pointing triangles), and polystyrene (circles).

The main result of this paper is twofold. First, we present experimental evidence (Fig. 2*E*) that the dewetting mechanism underlying the threshold criterion Eq. **3** does not capture the actual delamination threshold for highly bendable sheets—instead, the scaling Eq. **4** is most appropriate for the thinnest sheets studied. Second, we rationalize this observation by presenting a partial delamination model that describes curvature-induced delamination of highly bendable sheets and is strictly different from the dewetting mechanism of ref. 15. We show that partial delamination occurs by the formation of blisters that exploit the low energetic cost of bending to relieve strain and thereby suppress elastic energy in the sheet while maximizing its contact with the curved substrate. These blisters may be either small-amplitude rucks or large-amplitude folds, depending on the bendability of the sheet. Specifically, we identify a “highly bendable” parameter regime, where partial delamination is folds-based and the transition from a fully laminated state is given by Eq. **4**, and a “moderately bendable” parameter regime, where partial delamination is mediated by rucks. In the latter, the transition from a fully laminated state is described by a novel scaling relation that interpolates between the lower (compression-based) threshold Eq. **4** and the upper (dewetting-based) threshold Eq. **3**. This theory allows the experimental data points of Fig. 2*E* to be collapsed onto a master curve (Fig. 2*F*) and recovers the expected scaling regimes.

## 1. Raw Experimental Results

As a first indication that the energetic picture is not the full story, we present experimental results that interrogate the transition from smooth adhesion to delamination for circular sheets of radius *W* and thickness *t* and a sphere of radius *R*. In these experiments (*Materials & Methods* and *SI Appendix*), sheets with thickness *t* ∈ [100 nm, 104 μm] are deposited from floating on a water bath onto the sphere. After the system dries, the sheet is observed to either be smoothly adhered (represented by a filled point in Fig. 2*E*) or be partially delaminated (an open point in Fig. 2*E*).

As expected, the results of this experiment (summarized in Fig. 2*E*) show that above a critical radius ratio $\tilde{W}>{\tilde{W}}_{c}$, the sheet delaminates (modulo some imperfection-induced noise close to the transition). However, the experimental data shown in Fig. 2*E* do not show the scaling ${\tilde{W}}_{c}∼\beta^{1/4}$ predicted by the curvature-induced dewetting mechanism of Eq. **3**: the only plausibly power-law behavior that we observe appears to be ${\tilde{W}}_{c}\propto Y^{-1/2}∼\beta^{1/2}$, reminiscent of Eq. **4**, and this scaling is observed only in the sheets with the smallest values of *Y*.

## 2. The Onset of Blistering

### A. The Importance of Compression.

We begin our reexamination of the theory by taking a step back to consider the stress state within the elastic film as the sheet radius *W* changes, under the assumption that the sheet remains perfectly attached to the sphere and hence axisymmetric. In this case, the vertical deformation of the sheet is:

[5] $$\begin{array}{c} \zeta =-r^{2}/\left(2R\right), \end{array}$$

(from the parabolic approximation to the sphere’s surface, valid when *W*/*R* ≪ 1). The in-plane deformation and consequent stress within the laminated film are caused by the Gaussian curvature (∼1/*R*^2^), imparted by the out-of-plane deformation, as well as the radial tensile load *T* = *γ*_*su* − *v*_ applied by the substrate–vapor interfacial tension (17, 20).

The stress profile within the sheet can then be readily calculated from this displacement and has been presented in related work (16, 17). This calculation gives that the radial and hoop stresses within the sheet induced by the deformation of Eq. **5** are

[6] $$\sigma_{\mathrm{rr}}=\gamma_{su-v}+\frac{Y}{16R^{2}}\left(W^{2}-r^{2}\right),$$

[7] $$\sigma_{\theta \theta }=\gamma_{su-v}+\frac{Y}{16R^{2}}\left(W^{2}-3r^{2}\right).$$

We see that *σ*_*rr*_ >  0 throughout the sheet but also that the minimum value of the hoop stress, *σ*_*θθ*_^min^, is

[8] $$\begin{array}{c} \sigma_{\theta \theta }^{\text{min}}=\sigma_{\theta \theta }\left(W\right)=\gamma_{su-v}-\frac{YW^{2}}{8R^{2}}=Y\left(\beta \frac{\gamma_{su-v}}{\Gamma }-\frac{{\tilde{W}}^{2}}{8}\right), \end{array}$$

while the radial displacement of the sheet’s edge, *u*_*r*_^axi^(*W*), satisfies

[9] $$\begin{array}{c} \frac{u_{r}^{\text{axi}}\left(W\right)}{W}=\frac{\sigma_{\theta \theta }\left(W\right)-\nu \sigma_{\mathrm{rr}}\left(W\right)}{Y}=\left(1-\nu \right)\beta \frac{\gamma_{su-v}}{\Gamma }-\frac{1}{8}{\tilde{W}}^{2}. \end{array}$$

Crucially, Eq. **8** determines the numerical prefactor in the scaling Eq. **4**, namely:

[10] $$\begin{array}{c} {\tilde{W}}_{\text{comp}}=2^{3/2}{\left(\frac{\gamma_{su-v}}{\Gamma }\right)}^{1/2}\beta^{1/2}, \end{array}$$

so that for $\tilde{W}>{\tilde{W}}_{\text{comp}}$, the hoop stress becomes compressive at the edge of the sheet, *σ*_*θθ*_^min^ <  0. As we discussed in the introduction, this scaling matches the experimentally determined critical sheet size at the onset of delamination for the thinnest elastic sheets, ${\tilde{W}}_{c}\propto Y^{-1/2}$, as shown in Fig. 2*E*. However, we also see that there is a numerical factor that depends on the different surface energies in the problem: In general, *γ*_*su* − *v*_/*Γ* ≠ 1 since the adhesion energy *Γ* = *γ*_*su* − *v*_ + *γ*_*sh* − *v*_ − *γ*_*su* − *sh*_ = *γ*_*su* − *v*_(1 + cos*θ*_*Y*_) (with *γ*_*sh* − *v*_ and *γ*_*su* − *sh*_ the sheet–vapor and substrate–sheet surface energies, respectively, and *θ*_*Y*_ an effective contact angle). For simplicity, we shall take *γ*_*su* − *v*_ = *Γ* in what follows, corresponding to *γ*_*sh* − *v*_ = *γ*_*su* − *sh*_ or *θ*_*Y*_ = *π*/2. (Versions of the main results that follow are recorded in *SI Appendix* for values *γ*_*su* − *v*_/*Γ* ≠ 1.)

**Fig. 3.**
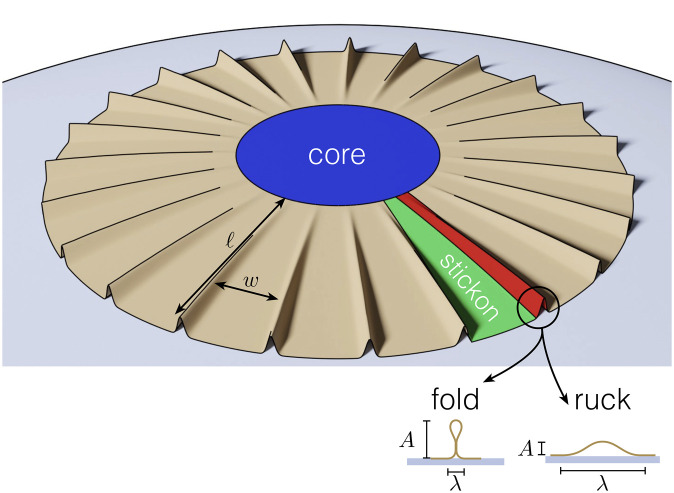
Schematic of a thin sheet attached to a sphere highlighting the adhered core region (blue), delamination blisters (red) and adhered ribbons, or “stickons,” between the blisters (green). Depending on the relative strength of bending and adhesion in this problem, the delamination blisters may form either folds or rucks (shown below the main figure). The length, ℓ, of the blisters, as well as the width *w* of a stickon region are also shown.

To understand how and when the appearance of compression affects the onset of delamination, we begin by considering the case in which the sheet has very little resistance to bending. In this case, a recent study of the one-dimensional analog problem (21) suggests that delamination blisters in highly bendable sheets take the form of ‘folds’: the amplitude *A* is large in comparison with the width of the blister *λ* (as shown in Fig. 3). A sheet coming into contact with an adhesive substrate suffers a discontinuity in its radius of curvature (22–24) proportional to the bendoadhesive length (25) ℓ_*ba*_ = (*B*/*Γ*)^1/2^ where *B* ∝ *Et*^3^ is the bending stiffness of the sheet. (Note that, in accord with the proposed terminology that distinguishes “elastocapillary” and “bendocapillary” lengths (26), we call this length “bendoadhesive” rather than “elastoadhesive” used by ref. 25.) Since a high-amplitude fold is largely flat (Fig. 3), its width is determined by the radius of curvature at the contact, i.e., *λ* ∼ ℓ_*ba*_. In the axisymmetric case, we similarly expect folds to be energetically favorable when $\tilde{W}>{\tilde{W}}_{\text{comp}}$ and the bending stiffness is sufficiently small. We therefore define a third dimensionless group, that depends on the bending modulus

[11] $$\begin{array}{c} \epsilon =\frac{B}{\Gamma R^{2}}={\left(\frac{\ell_{\mathrm{ba}}}{R}\right)}^{2}. \end{array}$$

Here, *ϵ* is a dimensionless bending stiffness and *ϵ*^−1^ characterizes the degree of “bendability” of the adhesive film (18): sheets for which *ϵ*^−1^ ≫ 1, or *R* ≫ ℓ_*ba*_, are readily bent to a radius of curvature *R* by adhesive forces.

Assuming that the blisters in a sheet delaminating from a sphere are radially elongated and are hence locally one-dimensional, adopting a similar fold shape, we now turn to understanding when such folds are expected and how many of them should form. This will also allow us to perform a consistency check of the fold ansatz a posteriori and to make the notion of “bendable” more precise.

### B. The Formation of Folds.

The key feature of a fold is that the bending energy is localized close to the contact region and in the loop—the majority of the arclength of the material is simply uncurved (albeit vertical)—and so the bending energy of a single fold of extent ℓ in the radial direction (Fig. 3) is *B*ℓ/ℓ_*ba*_ = (*BΓ*)^1/2^ℓ. As a result the bending energy in *n* folds (all of radial length ℓ) is

[12] $$\begin{array}{c} U_{\text{bend}}^{\text{fold}}∼n{\left(B\text{Γ}\right)}^{1/2}l. \end{array}$$

As should be expected, $U_{\text{bend}}^{\text{fold}}$ penalizes the creation of more folds (and hence blisters); to minimize this energy, the system should have as few blisters as possible. However, forming a small number of blisters is expensive in terms of strain energy because the portion of the sheet that remains adhered still conforms to the sphere; if there are fewer blisters, the attached ribbon-like elements between the blisters (or ‘stickons’, Fig. 3) must be wider and so be more highly strained. The strain energy per unit area of a stickon of width *w* adhered to a sphere of radius of curvature *R* is *u*_rib_ ∼ *Yw*^4^/*R*^4^ (15, 27); assuming that stickons are wide compared to the blisters formed, *w* ≫ *λ*, (i.e., most of the sheet remains laminated to gain adhesion energy) we have that *w* ∼ *W*_*c*_/*n* close to onset. The total strain energy stored in these ribbons, *U*_strain_ = *u*_rib_ × *W*_*c*_ℓ, is

[13] $$\begin{array}{c} U_{\text{strain}}∼\frac{YW_{c}^{4}}{n^{4}R^{4}}\times W_{c}\ell . \end{array}$$

(Note that there is still a strained, fully laminated core region, but that this does not play a role in the selection of the number of blisters that are formed.)

As expected, the energy *U*_strain_ drives the system to have many blisters, thereby competing with the bending energy $U_{\text{bend}}^{\text{fold}}$ to determine the optimal number of folds

[14] $$\begin{array}{c} n_{\text{fold}}∼\frac{W_{c}}{R}{\left(\beta^{2}\epsilon \right)}^{-1/10}∼{\left(\beta^{3}/\epsilon \right)}^{1/10}, \end{array}$$

where we use the assumption that folds form in tandem with the emergence of compression, i.e.,

[15] $$\begin{array}{c} {\tilde{W}}_{c}\approx {\tilde{W}}_{\text{comp}}∼\beta^{1/2}, \end{array}$$

in the last equality in Eq. **14**.

To understand when this regime is expected experimentally, we note two conditions on the formation of folds. First, folds are distinguished by being much taller than their width, i.e., *A* ≫ *λ*. To estimate the amplitude of folds, we denote the sheet length to be wasted by these blisters by *Δ*_tot_, so that *A* ∼ *Δ*_tot_/*n*_fold_. The fold ansatz is therefore valid provided that *Δ*_tot_/*n*_fold_ ≫ ℓ_*ba*_ or

[16] $$\begin{array}{c} \frac{\Delta_{\text{tot}}}{R}\gg {\left(\epsilon^{4}\beta^{3}\right)}^{1/10}. \end{array}$$

Second, and simplifying further our analysis by considering the case *ν* = 0, we note that since folds form close to the onset of compression in the sheet, their total arclength, *Δ*_tot_, may be estimated as *Δ*_tot_ ∼ |*u*_*r*_^axi^(*W*)|. Recalling that the underlying assumption in the fold regime is that ${\tilde{W}}_{c}={\tilde{W}}_{\text{comp}}+\delta \tilde{W}$ with $\delta \tilde{W}\ll {\tilde{W}}_{\text{comp}}∼\beta^{1/2}$, we can estimate $|u_{r}^{\mathrm{axi}}\left(W\right)/W|∼{\tilde{W}}_{\text{comp}}\;\delta \tilde{W}$ from Eq. **9**, so that the inequality Eq. **16** becomes ${{\tilde{W}}_{\text{comp}}}^{2}\;\delta \tilde{W}\gg {\left(\epsilon^{4}\beta^{3}\right)}^{1/10}$ or

[17] $$\begin{array}{c} {\left(\epsilon^{4}\beta^{-7}\right)}^{1/10}\ll \delta \tilde{W}\ll \beta^{1/2}. \end{array}$$

(For other values of *ν*, a detailed calculation, given in *SI Appendix*, shows that Eq. **17** holds regardless.) The separation of scales in Eq. **17** is only possible if

[18] $$\begin{array}{c} \epsilon \ll \beta^{3}. \end{array}$$

(Note that to determine the conditions under which folds form it was not necessary to evaluate $\delta \tilde{W}$ explicitly, and we hence have not needed to calculate *Δ*_tot_ at all!)

Eq. **18** makes precise our earlier statements that folds are expected when the bending stiffness of the sheet is sufficiently small: the appearance of folds requires *ϵ* ≪ *β*^3^ ⋘ 1. We note also from Eq. **14** that this high bendability regime corresponds automatically to a large number of folds—just as a large number of wrinkles is associated with the small cost of bending and, consequently, little resistance to compression, this is also the case for folds.

When *ϵ* ≳ *β*^3^, the fold ansatz used above is no longer self-consistent since it gives rise to folds for which the typical slope *A*/*λ* ≲ 1. If the slope of the delamination structures formed with *ϵ*/*β*^3^ ≫ 1 were indeed small, they would be more akin (28–30) to rucks in rugs (31, 32), rather than the folds we have considered so far. We therefore turn to consider rucks.

### C. The Formation of Rucks.

The simplicity of the fold case stems from the separate role of mechanics and geometry in their construction: the product *A* ⋅ *n*_fold_ of the amplitude and number of folds is given by the arclength *Δ*_tot_*that is to be wasted* (which is imposed by confining the sheet onto a sphere), whereas the fold’s width is determined by an energetic balance (which leads to *λ* ∼ ℓ_*ba*_, independently of *Δ*_tot_). Unlike a fold, a small-slope ruck of height *A* and width *λ* ≫ *A* does not exhibit such a “decoupling” of geometry and mechanics. Approximating a ruck by the sinusoidal profile $\zeta \left(x\right)\approx \frac{1}{2}A[1+\cos\left(2\pi x/\lambda \right)]$, the arclength “wasted” by the ruck is $\Delta =\int_{-\lambda /2}^{\lambda /2}({\left[1+\zeta \prime {\left(x\right)}^{2}\right]}^{1/2}-\;1)\;\text{d}x∼A^{2}/\lambda$. However, for a ruck, *A* and *λ* cannot be chosen independently: A local force balance (22) or variational arguments (23, 24) reveals that these two lengths are constrained by the requirement that the radius of curvature of the blister at the delamination point matches the bendoadhesive length (22, 23, ℓ_*ba*_. This condition gives that *λ*^2^/*A* ∼ ℓ_*ba*_, which, combined with the wasted length constraint, gives

[19] $$\begin{array}{c} \lambda ∼\Delta^{1/3}\ell_{\mathrm{ba}}^{2/3},\;A∼\Delta^{2/3}\ell_{\mathrm{ba}}^{1/3}. \end{array}$$

These scalings have been derived previously in the context of the “sticky elastica” problem (24) in which a blister with a given wasted length *Δ* is formed and its dimensions are measured. Unlike the sticky elastica problem, however, the length to be wasted in each delamination blister is not controlled here: While Eq. **9** implies a global constraint for the total amount of length that must be wasted, *Δ*_tot_, there is, as yet, no constraint on the number of blisters that will form, each wasting a length *Δ* = *Δ*_tot_/*n*.

Anticipating that the ruck ansatz will be the appropriate (i.e., self-consistent) one for moderately bendable sheets (which, based on the fold case, should correspond to *ϵ*/*β*^3^ ≫ 1), we also expect that the threshold sheet size for delamination will be well beyond the critical size at which a hoop compression first develops, i.e., that ${\tilde{W}}_{c}\gg \beta^{1/2}$. From Eq. **9**, we therefore have that $|u_{r}\left(W_{c}\right)|∼R{\tilde{W}}_{c}^{3}$. Our working hypothesis is that, upon delamination, this excess length is all wasted by buckling, so that

[20] $$\begin{array}{c} \Delta_{\text{tot}}\approx u_{r}\left(W_{c}\right)∼R{\tilde{W}}_{c}^{3}. \end{array}$$

We therefore repeat the energetic balance argument that allowed us to determine the number of folds in the highly bendable limit: as before, bending energy seeks to form as few rucks as possible, while the strain in the laminated portions of the sheet drives it to form as many as possible. The important difference with the earlier analysis of folds is that the bending energy within a ruck is distributed all along its arclength; we therefore have that the bending energy of all *n* rucks is

[21] $$\begin{array}{c} U_{\text{bend}}^{\text{ruck}}∼n\times B{\left(A/\lambda^{2}\right)}^{2}\times \lambda l∼\text{Γ}n^{2/3}{\text{Δ}}_{\text{tot}}^{1/3}l_{ba}^{2/3}l. \end{array}$$

The strain energy stored in the laminated portions of the sheet, the stickons, is (in scaling terms) independent of the form that the delamination blisters take, so that Eq. **13** still holds. We can then determine that the optimal number of rucks is

[22] $$\begin{array}{c} n_{\text{ruck}}∼{\left(\beta^{3}\epsilon \right)}^{-1/14}\frac{W_{c}}{R}{\left(\frac{\Delta_{\text{tot}}}{W_{c}}\right)}^{-1/14}∼{\left(\beta^{3}\epsilon \right)}^{-1/14}{\tilde{W}}_{c}^{6/7}. \end{array}$$

At this stage, an important difference with the fold case emerges: we do not a priori know the value of $\tilde{W}$ at which ruck-like delamination blisters will emerge, and so we cannot use Eq. **22** to determine *n*_ruck_ in terms of *ϵ* and *β*. Instead, we determine the threshold ${\tilde{W}}_{c}$ by the standard buckling criterion, i.e., by equating the residual hoop compression in the delaminated state, *σ*_res_ ∼ *B*/*λ*^2^ with *λ* given by Eq. **19** (ref. 33) and the hoop compression of the axisymmetric (laminated) state, $|{\sigma_{\theta \theta }}^{\min}|∼Y{\tilde{W}}^{2}$, which we evaluate from Eq. **8**, recalling that ${\tilde{W}}_{c}\gg \beta^{1/2}$ for rucks. This buckling criterion yields the delamination-into-rucks threshold:

[23] $$\begin{array}{c} {\tilde{W}}_{c}∼{\left(\beta^{3}\epsilon \right)}^{1/12}. \end{array}$$

We note that the threshold in Eq. **23** reproduces the fold scaling Eq. **15**, i.e., ${\tilde{W}}_{c}∼\beta^{1/2}$, as *ϵ*/*β*^3^ ↘ 1, while for *ϵ*/*β*^3^ ≫ 1 the onset for delamination into rucks occurs at ${\tilde{W}}_{c}\gg \beta^{1/2}$. Similarly, we note that when *ϵ* = *O*(1), Eq. **23** recovers the prediction of the upper bound Eq. **3** that delamination is favorable when $\tilde{W}≳\beta^{1/4}$.

As a final consistency check, we note that the aspect ratio of delamination rucks at onset is

[24] $$\begin{array}{c} \frac{A}{\lambda }∼{\left(\frac{\Delta }{\ell_{\mathrm{ba}}}\right)}^{1/3}∼{\left(\frac{\Delta_{\text{tot}}/n}{\ell_{\mathrm{ba}}}\right)}^{1/3}∼{\left(\frac{\beta^{3}}{\epsilon }\right)}^{1/12}. \end{array}$$

As expected, for *ϵ* ≫ *β*^3^, the aspect ratio of the blisters *A*/*λ* ≪ 1 and our assumption of small-slope rucks (rather than large-slope folds) is indeed self-consistent.

## 3. Experimental Measurements of Onset

Combining the two predictions Eq. **15** and Eq. **23**, we have that the critical radius at the onset of delamination is predicted to scale with *ϵ* and *β* according to

[25] $$\begin{array}{c} {\tilde{W}}_{c}∼\left\{\begin{array}{cc} \beta^{1/2} & \epsilon \ll \beta^{3},\\ {\left(\epsilon \beta^{3}\right)}^{1/12} & \beta^{3}\ll \epsilon \ll 1. \end{array}\right. \end{array}$$

We note that these two results may alternatively be written:

[26] $$\begin{array}{c} \frac{{\tilde{W}}_{c}}{\beta^{1/2}}∼\left\{\begin{array}{cc} 1 & \epsilon /\beta^{3}\ll 1,\\ {\left(\epsilon /\beta^{3}\right)}^{1/12} & \epsilon /\beta^{3}\gg 1. \end{array}\right. \end{array}$$

Since this form presents different results in terms of the effective bendability of the sheets, *ϵ*/*β*^3^, it is a useful one for reconsidering the experimental data presented in Fig. 2*E*, to which we now turn.

As noted already in the introduction, a first experimental indication of the importance of partial delamination is exhibited by the data for the thinnest sheets shown in Fig. 2*E* and is consistent with the *ϵ*-independent scaling of the delamination threshold for sufficiently small *ϵ* (first line in Eq. **25**). To test the model predictions beyond this ultrathin regime, Eq. Fig. 2*F* shows the same data as Fig. 2*E* replotted with the two axes rescaled according to Eq. **26**. Since the sphere’s curvature as well as the elastic moduli and thicknesses of the sheets are known at high precision, the only fitting parameter used in Fig. 2 is *γ*_*su* − *v*_ = *Γ*, which has been assumed to be the same for all sheet materials used. In fact, while *γ*_*su* − *v*_ is constant in our experiments (which use the same polycarbonate spheres), *Γ* is expected to vary slightly for the different sheet materials. Our theory suggests that *γ*_*su* − *v*_ controls the plateau for highly bendable sheets, while *Γ* controls the collapse in the moderately bendable regime; while we might therefore expect some imperfections in the collapse in this regime, our experimental results do not suggest significant variation in *Γ* between materials.

Beyond the experimental support for the predicted scaling exponent in the ruck-based regime Eq. **23**, we emphasize that the mere collapse of data exhibited in Fig. 2*F* confirms that the threshold size for curvature-induced delamination of thin films is governed by an “effective bendability” parameter, *ϵ*/*β*^3^ ∝ *BY*^3^/*Γ*^4^*R*^2^, that depends in a rather nontrivial manner on both bending and stretching moduli of the sheet, as well as the adhesion parameter and substrate curvature. We also note that the plateau in ${\tilde{W}}_{c}$ corresponds to the power-law regime evident in Fig. 2*E* but is now reduced to a smaller relative interval because the rescaling used in Fig. 2*F* highlights instead the *ϵ*-dependent threshold of the rucks-based regime. Notably, the plateau is observed with *ϵ*/*β*^3^ finite but large (*ϵ*/*β*^3^ ≲ 10^2^), rather than strictly *ϵ*/*β*^3^ ≪ 1; this indicates the presence of a large numerical prefactor that cannot be determined by our scaling analysis.

**Fig. 4.**
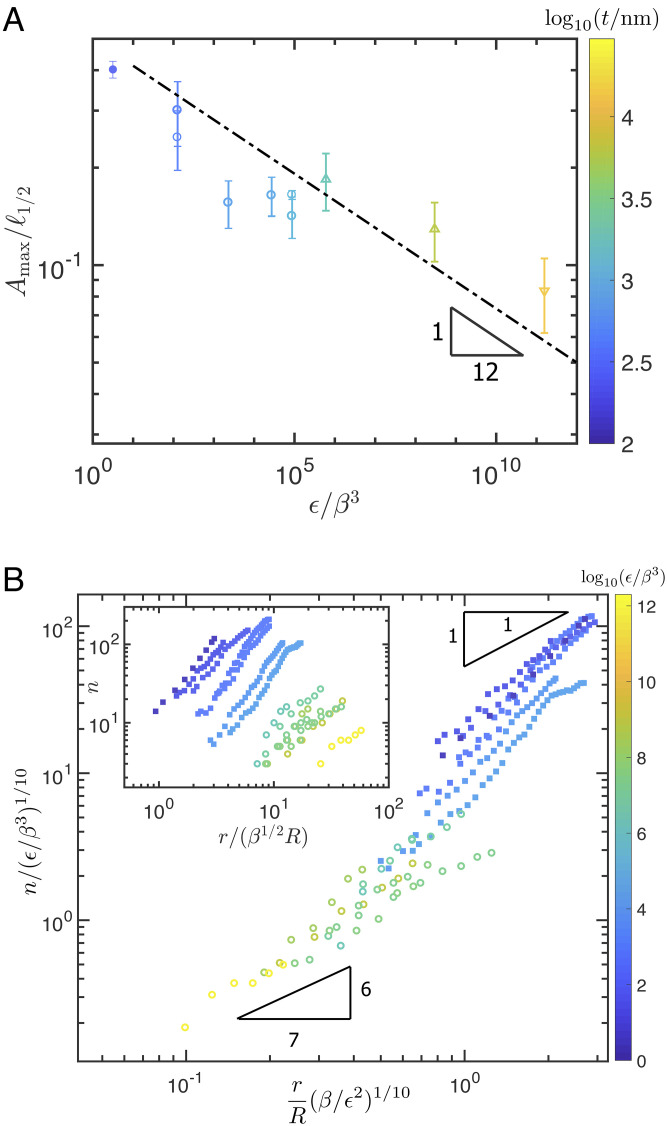
Properties of delamination blisters beyond onset. (*A*) The experimentally measured aspect ratio of delamination blisters in the ruck regime, showing that experiments are consistent with the scaling for aspect ratio of rucks given in Eq. **24** and shown by the dash-dotted line. Here, aspect ratios are determined from optical interference (open points) and profilometry (closed point) which gives the maximum amplitude, *A*_max_, and the full width at half maximum, ℓ_1/2_; symbols are used as in Fig. 2 to show sheet type and thickness. (*B*) The spatial distribution of the fold number, *n*. Here, experiments are performed with PS sheets (filled symbols) and PC sheets (open symbols) with the color bar encoding the value of *ϵ*/*β*^3^. According to the theory, Eq. **29**, when plotted in this way bluer/darker points should lie closer to the linear scaling expected for folds, while yellower/lighter points should lie closer to the 6/7 exponent expected for rucks. The experimental results are largely consistent with the theory and suggest that the exponent of spatial variation is larger for smaller *ϵ*/*β*^3^. Moreover, the rescaled data show a reasonable collapse when compared with the raw data (inset).

The features of the experimental data as plotted in Fig. 2*F* are nontrivial tests of the presented theoretical picture. However, another useful comparison with experiments comes from the aspect ratio of the delamination blisters, measured near the edge of the sheet, in the ruck regime. (This was measured in the case of rucks using an optical interference technique, described in *SI Appendix*, that cannot resolve the large slopes of folds.) These measurements are presented in Fig. 4*A* and are also consistent with Eq. **24** and hence the theoretical picture as a whole. Note, however, that these experiments were not performed “at” threshold but at some distance beyond it. It is therefore natural to consider the problem of what happens beyond the initial threshold a little further.

## 4. Beyond Threshold: Spatial Structure of Folds

The argument so far has focussed on the behavior at the onset of the delamination instability. However, similar arguments can be used to understand what the desired spatial structure of the fold and ruck pattern might be beyond threshold. Provided that the radial position *r* is large compared to the blister width, *r* ≫ *λ*, and that the cost of changing the number of delamination blisters may be neglected, the same arguments used to derive the number of blisters at onset can be repeated with *W*_*c*_ replaced by *r*. (Predictions for the spatial variation of other properties of the blister pattern, such as the blister amplitude and width could then be inferred from these results together with the wasted length constraint.) By making this substitution in Eq. **13** and minimizing the combination of this and the bending energy Eq. **12** by varying *n*(*r*), we find that

[27] $$\begin{array}{c} n_{\text{fold}}∼\frac{r}{R}{\left(\beta^{2}\epsilon \right)}^{-1/10}. \end{array}$$

In the ruck case, repeating the replace and minimization procedure on Eqs. **13**, **20** and **21** gives

[28] $$\begin{array}{c} n_{\text{ruck}}(r)∼{\left(\epsilon \beta^{3}\right)}^{-1/14}{\left(\frac{r}{R}\right)}^{6/7}. \end{array}$$

(As should be expected of these generalizations, Eqs. **27** and **28** reduce to Eqs. **14** and **22**, respectively when $r\to {\tilde{W}}_{c}R$.)

We see that the evolution of mode number of instability with radial position, *r*, depends on whether that instability takes place via folds or rucks—compare the linear scaling with radial position *r* of Eq. **27** and the sublinear scaling with *r* of Eq. **28**. These two scalings can be written in terms of common variables as:

[29] $$\begin{array}{c} n{\left(\frac{\beta^{3}}{\epsilon }\right)}^{1/10}∼\left\{\begin{array}{cc} \frac{r}{R}{\left(\frac{\beta }{\epsilon^{2}}\right)}^{1/10} & \epsilon \ll \beta^{3},\\ {\left[\frac{r}{R}{\left(\frac{\beta }{\epsilon^{2}}\right)}^{1/10}\right]}^{6/7} & \epsilon \gg \beta^{3}. \end{array}\right. \end{array}$$

The predictions of Eq. **29** are compared with experimental results in Fig. 4*B*. Points with larger values of *ϵ*/*β*^3^ (paler/yellower points) are consistent with the sublinear scaling expected for rucks, while those with smaller values of *ϵ*/*β*^3^ (darker/bluer points) are consistent with the linear scaling expected for folds. This consistency with the prediction of Eq. **29** suggests that the energetic cost of changing the number of folds or rucks is negligible. We note that a similar feature has been shown to characterize also wrinkle patterns, whereby a “microscopic” wavelength is determined by local energy considerations, and its value may vary smoothly across a confined sheet (33). The collapse of our experimental data shown in the main portion of Fig. 4*B* shows two further noteworthy features: First, while the exponents of the two behaviors are ostensibly close (6/7 versus unity), the associated prefactors seem to be very different, separating the ruck and fold behaviors; second, the steep transition region between the two behaviors suggests that the central regions of highly bendable sheets may form rucks, rather than folds.

## 5. Conclusions

We have shown that the threshold size ${\tilde{W}}_{c}$ above which a bendable sheet delaminates from an adhesive spherical substrate is not determined merely by the adhesion strength and stretching modulus, as has been supposed previously (3, 15, 34, 35). Instead, there is a strong effect of the bending modulus underlying the nontrivial dependence of ${\tilde{W}}_{c}$ on the two-dimensionless groups *β* and *ϵ* that is given in Eq. **25**.

In particular, we have shown that there are two modes through which partial delamination allows the sheet to expel excess area, relieving strain while maximizing contact area. When *ϵ*/*β*^3^ ≪ 1 (or, equivalently, $\Gamma \gg Y\sqrt{t/R}$), the sheet delaminates from the sphere by forming many large-slope folds, while for *ϵ*/*β*^3^ ≫ 1 (i.e., $\Gamma \ll Y\sqrt{t/R}$), delamination occurs via small-slope rucks. Despite the differences in the morphology of partial delamination patterns, in both cases, the onset of instability occurs for sheets that are significantly smaller than the previously presented “upper bound” ${\tilde{W}}_{\text{dewet}}∼\beta^{1/4}$, which was obtained by incorporating the total elastic energy into the standard dewetting criterion for two phases (15).

The key difference between the previously identified upper bound on the size of sheets that can conform perfectly to a sphere and our results arises from the fact that ${\tilde{W}}_{\text{dewet}}$ does not take into consideration the inhomogeneous, anisotropic distribution of strain in the laminated state, and the consequent presence of compression even for rather small sheet sizes ${\tilde{W}}_{\text{comp}}<\tilde{W}\ll {\tilde{W}}_{\text{dewet}}$, where ${\tilde{W}}_{\text{comp}}∼\beta^{1/2}$, Eq. **15**. Indeed, the emergence of fold and ruck patterns follows directly from the tendency of a thin sheet to maximize adhesion by “trading” an energetically expensive strain with energetically cheap bending. This principle is analogous to the one underlying wrinkle patterns in sheets confined to curved deformable substrates (19, 33, 36, 37); the morphological complexity of partial delamination patterns in comparison to their wrinkle counterparts follows from the binary (nonanalytic) nature of the adhesion energy, which is insensitive to the deflection amplitude.

The collapse of the data of Fig. 2*E* that is exhibited in Fig. 2*F* includes measured threshold sizes of circular films made of various materials and a broad range of thicknesses and therefore indicates the robustness of the partial-delamination model. To further stress this point, we highlight two key assumptions underlying our model. First, a central, yet thus far implicit, assumption is that deformation of the sheet is determined by minimization of the system’s energy (composed of interfacial and elastic contributions). While we cannot totally rule out the presence of irreversible processes, most notably sheet–substrate friction, which would hinder the approach of the system to its optimal configuration, the agreement with the predicted scaling laws suggests that any deviations from the predicted partially delaminated energy minimum are small (in comparison to the energetic gain relative to a fully laminated state). Second, another assumption is the rigidity of the substrate. Indeed, it has been argued that if a spherical substrate is sufficiently deformable, strain in the laminated sheet may be reduced considerably by flattening the substrate, rather than the sheet delaminating from the substrate, pushing the delamination threshold beyond Eq. **3**. Crucially, the critical Young’s modulus of a substrate below which such a nonrigid response is expected can be estimated (17) as $E_{\mathrm{subs}}^{\mathrm{crit}}∼\Gamma^{3/4}Y^{1/4}/t∼10\;\mathrm{MPa}$, which is at least two orders of magnitude softer than the modulus of the substrates used experimentally here.

We have seen that sheets do not conform perfectly to a spherical substrate once their lateral size increases beyond a limit. In applications that involve a sheet (approximately) conforming to a doubly curved substrate, it is nevertheless of interest to determine the extent to which some delamination is minor: Does the sheet remain largely conformed to the substrate? We therefore define the “conformability” of the sheet to the sphere, 𝒞(*r*), as the proportion of the infinitesimal annulus [*r*, *r* + *δr*] that is covered by the sheet, i.e.,

[30] $$\begin{array}{c} C\left(r\right)=1-\frac{n(r)\lambda (r)}{2\pi r}. \end{array}$$

Using the results already given we have that the delaminated proportion, D(r)=1−C(r) is

[31] $$\begin{array}{c} D\left(r\right)∼\left\{\begin{array}{cc} {\left(\epsilon^{2}/\beta \right)}^{1/5} & \epsilon \ll \beta^{3}\ll 1,\\ {\left(\epsilon^{2}/\beta \right)}^{1/7}{\left(r/R\right)}^{4/7} & \beta^{3}\ll \epsilon \ll 1. \end{array}\right. \end{array}$$

The above expressions show three desirable features of the emergence of folds in highly bendable sheets for uniform conformation: C→1 in the (singular) infinite bendability limit *ϵ* → 0 (i.e., conformation becomes asymptotically perfect in this limit), D(r) is independent of *r*, and, throughout this regime, D≲β. In contrast, the conformation obtained by rucks is nonuniform and less effective in comparison to folds.

We now briefly consider the adhesion of graphene to a spherical substrate, as might be desired in a Surface Force Apparatus, for example. Taking values typical for graphene of *Y* ∼ 400 N m^−1^ (38) and bending energy *B* ∼ 1 eV ∼ 10^−19^ J (39), together with a radius of curvature typical of the Surface Force Apparatus, *R* ∼ 1 cm, and an adhesive energy *Γ* ∼ 0.1 N m^−1^, we find that *β* ∼ 2 × 10^−5^, *ϵ* ∼ 10^−14^ so that *ϵ*/*β*^3^ ∼ 1, which is sufficient for graphene to lie within the high bendability regime. In particular, we expect that ${\tilde{W}}_{c}∼\beta^{1/2}∼5\times 10^{-3}$: sheets of graphene adhered to a hemisphere of radius of curvature 1 cm will only adhere smoothly if their radius *W* ≲ 50 μm. This is a significantly more stringent constraint on the adhesion of graphene to doubly curved surfaces than would have been the case in the picture presented by the upper bound (15), ${\tilde{W}}_{\text{dewet}}≃700\;\mu m$. It may also explain why applications in which graphene spontaneously adheres to curved substrates have been observed to form delamination blisters (4) with a morphology similar to the rucks and folds studied here.

The theory and experiments presented in this paper show that the anisotropic and inhomogeneous response of thin elastic sheets to compression manifests itself in both the macroscopic and the microscopic behaviors of such sheets: Both large-scale features such as the threshold between delamination and smooth adhesion (not to mention conformability) and small-scale features like the number of blisters can only be understood through a proper understanding of these different responses. Moreover, this understanding points the way to better control of conformability in scenarios ranging from the humble band-aid to industrial coatings and beyond.

## Materials and Methods

Polystyrene sheets were fabricated by spin-coating polystyrene dissolved in toluene, while sheets of Polyvinylsiloxane were obtained by spin-coating Z-Dupe (Henry Schein); sheets of other materials were obtained commercially. Discs were then cut from these sheets and floated at the surface of a bath of water; after placing a an Acrylic/Polycarbonate spherical cap beneath the floating sheet, the water bath was gradually drained until the sheet was deposited on the spherical cap. The system was then allowed to dry for at least 10 min before the sheet was inspected to determine whether delamination had taken place and the properties of any delamination blister pattern were measured. Further details of the experimental procedure may be found in *SI Appendix*.

## Supplementary Material

Appendix 01 (PDF)

## Data Availability

All study data are included in the article and/or *SI Appendix*.
