# Supplementary material for: Delamination from an adhesive sphere: Curvature-induced dewetting versus buckling
Source: Proc Natl Acad Sci U S A. 2023 Mar 17;120(12):e2212290120. doi: 10.1073/pnas.2212290120 (PMC10041104; doi:10.1073/pnas.2212290120)
Supplement: Supplementary file 1 — Appendix 01 (PDF) [file pnas.2212290120.sapp.pdf]

Supplementary Information for

Delamination from an adhesive sphere: Curvature–induced dewetting versus buckling

F. Box, L. Domino, T. Outerelo Corvo, M. Adda-Bedia, V. Démery, D. Vella and B. Davidovitch

Corresponding Author name.
E-mail: dominic.vella@maths.ox.ac.uk

This PDF file includes:

Supplementary text
Figs. S1 to S2
SI References

Supporting Information Text

This Supplementary Information file contains further details of the theoretical picture presented in the main paper in §1, including the detailed calculation of the length available to waste in the formation of folds. §2 provides details of the experimental methods.

1. Further theory details

A. General calculation of length wasted in folds. To show that folds can only occur when $\epsilon \ll \beta^3$ (eqn (18) of the main text) it was assumed that the Poisson ratio $\nu = 0$. This simplified the calculation of the length that must be wasted by fold formation, Δ_{tot}, but the result actually holds for all values of ν. To show this, we consider here how the argument is modified for non-zero Poisson ratios.

The key observation is that for highly bendable sheets, the formation of delamination folds relaxes the compressive stress, *i.e.* $\sigma_{\theta\theta} \approx 0$ (1). (More precisely, the maximal level of the compressive stress, $|\min(\sigma_{\theta\theta})|$, accommodated by the axisymmetric, unbuckled state, vanishes as $\epsilon \to 0$.) As such, the linear constitutive relation (the tensor equation equivalent of Hooke's law) gives the azimuthal (hoop) strain at the edge of the sheet at threshold as:

$$\epsilon_{\theta\theta}|_{r=W} = \frac{\sigma_{\theta\theta} - \nu\sigma_{rr}}{Y} \approx -\nu\frac{\sigma_{rr}(W)}{Y} = -\nu\beta. \qquad \text{[S1]}$$

The azimuthal strain may also be expressed in terms of the radial and normal displacements (u_r and ζ, respectively) as:

$$\epsilon_{\theta\theta} = \frac{u_r}{r} + \frac{1}{2r^2}\left(\frac{\partial\zeta}{\partial\theta}\right)^2. \qquad \text{[S2]}$$

We therefore combine Eqns (S1) and (S2) with the assumption that in the fold (high bendability) limit, the radial displacement $u_r(W)$ does not change significantly from its value in the axisymmetric setup (*i.e.* that sufficiently close to threshold, $u_r(W) \approx u_r^{\text{axi}}(W)\cdot\left[1 + O(\epsilon^a)\right]$ for some $a > 0$) to calculate the length to be wasted Δ_{tot} by folds, $\Delta_{\text{tot}} = \int_0^{2\pi} \frac{1}{2r^2}\left(\frac{\partial\zeta}{\partial\theta}\right)^2 \, W\mathrm{d}\theta$. We find that

$$-\nu\beta = \epsilon_{\theta\theta}|_{r=W} = \frac{u_r(W)}{W} + \frac{\Delta_{\text{tot}}}{W} \approx \frac{u_r^{\text{axi}}(W)}{W} + \frac{\Delta_{\text{tot}}}{W} \qquad \text{[S3]}$$

and hence

$$\frac{\Delta_{\text{tot}}}{W} \approx -\frac{u_r^{\text{axi}}(W)}{W} - \nu\beta = \left|\frac{u_r^{\text{axi}}(W)}{W} + \nu\beta\right|. \qquad \text{[S4]}$$

Recalling that in the fold regime we anticipate that the critical size for the fold instability is close to that at which compression first occurs, i.e. $W_c = W_{\text{comp}} + \delta W$ with $\delta W/W_{\text{comp}} \ll 1$, and using Eq. (10) of the main text we then have

$$\frac{\Delta_{\text{tot}}}{W} \approx \left|\frac{u_r^{\text{axi}}(W)}{W} + \nu\beta\right| = \left|(1-\nu)\beta - \frac{1}{8}\tilde{W}^2 + \nu\beta\right| = \left|\beta - \frac{1}{8}\left(2\sqrt{2}\beta^{1/2} + \delta\tilde{W}\right)^2\right| \approx \beta^{1/2}\delta\tilde{W}/\sqrt{2}. \qquad \text{[S5]}$$

Eq. (S5) is identical to the scaling relationship used to derive eqn (18) of the main text under the assumption that $\nu = 0$; as a result the main conclusion, eqn (18) of the main text, holds irrespective of Poisson ratio.

B. Leading order correction of w_c/R when $\epsilon/\beta^3 \ll 1$. The above paragraph highlighted the central role of the compressive stress in the analysis of the highly bendable regime. Namely, it is the hoop component of the axisymmetric stress, $\sigma_{\theta\theta} < 0$, that is being suppressed upon delamination, rather than the corresponding strain $\epsilon_{\theta\theta}$ (which may retain a finite value $\epsilon_{\theta\theta} \approx -\nu\beta$, even after delamination). Pushing this observation further, we can improve our characterization of the threshold value to delamination folds by equating the maximal compression in the axisymmetric state, $\sigma_{\theta\theta}^{\text{axi}}(r = W)$, with the residual compressive stress in the folded state, $\sigma_{\theta\theta}^{\text{fold}}$. The former is expressed using Eqs (8) and (10) of the main text together with $(\tilde{W}_c/\tilde{W}_{\text{comp}}) - 1 \ll 1$ to give

$$\sigma_{\theta\theta}^{\text{axi}}(W) \sim -Y\tilde{W}_{\text{comp}}\cdot(\tilde{W}_c - \tilde{W}_{\text{comp}}). \qquad \text{[S6]}$$

The latter, $\sigma_{\theta\theta}^{\text{fold}}$, is evaluated via the residual (unavoidable) compression in a "stickon": $\sigma_{\theta\theta}^{\text{fold}} \sim -Y(w/R)^2 \sim -Yn(W)^{-2}\beta$, where we have used that the width of the delaminated zone is negligible, *i.e.* $\lambda(r) \ll r$, to estimate the width of each stickon as $w \sim W/n$. Using Eq. (14) of the main text to estimate n, we therefore find that:

$$\sigma_{\theta\theta}^{\text{fold}} \sim -Y(\epsilon\beta^2)^{1/5}. \qquad \text{[S7]}$$

Combining Eqs. (S6) and (S7) we obtain:

$$\tilde{W}_c(\beta,\epsilon) \sim \tilde{W}_{\text{comp}}\cdot\left[1 + c(\epsilon/\beta^3)^{1/5}\right], \qquad \text{[S8]}$$

for some constant c. This determines, at a scaling level, the correction to Eq. (15) of the main text. We shall see below that this prediction is consistent with experimental results performed on the thinnest PS sheets.

C. The case $\Gamma \neq \gamma_{su-v}$. In the main text, we assumed for simplicity that the adhesion energy, $\Gamma = \gamma_{su-v} + \gamma_{sh-v} - \gamma_{sh-su}$, was identical to the substrate-vapor surface energy, i.e. $\gamma_{su-v} = \Gamma$ (or, equivalently, $\gamma_{sh-v} = \gamma_{sh-su}$). In general, this is not expected to be the case, and so we record here the theoretical results that are obtained by relieving this assumption. Crucially, the characteristic scale that governs the width of folds and rucks remains the bendoadhesive length $\ell_{ba} = (B/\Gamma)^{1/2}$ while the applied boundary tension is $\gamma_{su-v} \neq \Gamma$. We shall see that it is essentially only the properties of the onset of delamination in the highly bendable (fold) regime that change when $\Gamma \neq \gamma_{su-v}$. In particular, the expressions for the critical sheet size, $\tilde{W}_c$, and the number of folds observed at onset change with respect to the results reported in the main text.

The fold regime Since Γ is the adhesion energy, the energetic cost of forming n folds of length ℓ is precisely as in (12) of the main text, that is

$$U_{\text{bend}}^{\text{fold}} \sim n(B\Gamma)^{1/2}\ell, \qquad \text{[S9]}$$

while the strain energy at the onset follows (13), which can be written

$$U_{\text{strain}}^{\text{fold}} \sim \frac{Y\tilde{W}_c^5}{n^4}R\ell \sim \frac{Y\beta^{5/2}}{n^4}\left(\frac{\gamma_{su-v}}{\Gamma}\right)^{5/2}R\ell. \qquad \text{[S10]}$$

Here we have used the expression for $\tilde{W}$ at the onset of compression, given by eqn (10) of the main text, as the estimate of $\tilde{W}_c$. Namely, the onset of compression is governed by the tensile load γ_{su-v} at the edge of the laminated film, rather than by the adhesion energy Γ (2). Balancing these two expressions, we find that the predicted number of folds at onset as

$$n_{\text{fold}} \sim \left(\frac{\beta^3}{\epsilon}\right)^{1/10}\left(\frac{\gamma_{su-v}}{\Gamma}\right)^{1/2}. \qquad \text{[S11]}$$

(We note that the dependence on the ratio of surface energies is relatively weak.)

Equation Eq. (S11) is derived on the basis that the blister has the form of a large-amplitude fold, and hence that $A \gg \lambda \sim \ell_{ba}$. The analog of eqn (16) of the main text is then that $\Delta_{\text{tot}}/R \gg (\beta^3\epsilon^4)^{1/10}(\gamma_{su-v}/\Gamma)^{1/2}$. We also have that $\Delta_{\text{tot}}/R \sim \tilde{W}_c^2\delta\tilde{W}$ so that (17) of the main text becomes

$$\left(\frac{\gamma_{su-v}}{\Gamma}\right)^{-1/2}(\epsilon^4\beta^{-7})^{1/10} \ll \delta\tilde{W} \ll \left(\frac{\gamma_{su-v}}{\Gamma}\right)^{1/2}\beta^{1/2} \qquad \text{[S12]}$$

and hence the condition for the ruck ansatz (eqn (18) of the main text) becomes

$$\frac{\epsilon}{\beta^3} \ll \left(\frac{\gamma_{su-v}}{\Gamma}\right)^{5/2}. \qquad \text{[S13]}$$

Here, we note that the dependence on the ratio of surface energies, γ_{su-v}/Γ, is relatively strong. We speculate that this dependence may explain why the plateau associated with the fold regime is observed even when the ratio ϵ/β^3 is on the order of $10-100$.

Beyond onset, the calculation above changes so that the strain energy $U_{\text{strain}}^{\text{fold}} \sim \ell Y r^5/(n^4R^4)$. When balancing this with the bending energy, we again recover eqn (27) of the main text.

The ruck regime In the calculation for the ruck regime, the value of $\tilde{W}$ at which compression first appears is not relevant. As such the results from the main text, eqns (22), (23) and (27) remain valid.

2. Experimental Methods

A. Sheet fabrication and description. Polystyrene (PS) sheets were fabricated by spin-coating PS (Goodfellow, Cambridge) dissolved in toluene onto glass slides. The thickness of the resulting solid PS sheets was measured using interferometry (Film Metrics F20), and discs of known radius were then cut from the sheet and floated on a bath of tap water. Here, results are presented for sheet thicknesses 120 nm $\leq t \leq 1.55$ μm, with a typical variation of 20 nm within each film. The highest bendability sheets were Polyvinylsiloxane sheets, obtained by spin-coating Z-Dupe (Henry Schein).

To reach larger thicknesses (lower bendability), pre-fabricated sheets of other glassy materials were purchased from Goodfellow (Cambridge, UK). In particular, sheets of Polycarbonate (PC) with $t = 2$ μm, 6 μm, and Polyimide (PI) with $t = 13$ μm, 25 μm were used.

The geometrical properties of the system (sheet radius W, thickness t and sphere radius of curvature R) were all known or measured as previously described; similarly the Young's modulus of the sheets is used as reported from the literature, i.e. $E = 3.4$ GPa (PS) from (3), and $E = 2.35$ GPa (Polycarbonate) and $E = 2.9$ GPa (Polyimide) from Goodfellow data sheets. $E = 663 \pm 6$ kPa was measured for Polyvinylsiloxane sheets by uniaxial, compressive testing a sample on an Instron 3345 structural testing system.

The only unknown in our system is the dry adhesion energy between the sheet and the sphere. This cannot be measured directly using standard blister tests because of the extremely thin sheets used. Instead, we use the behaviour with the very smallest values of ϵ/β^3 (where the plateau in $\tilde{W}_c$ is predicted to occur) to fit $\Gamma \approx 0.45$ Nm^{-1}; more details of this fitting procedure are given in §2.C. (It is only possible to measure Γ directly for the thinnest PS and PVS sheets, since these reach the smallest values of ϵ/β^3; we therefore use the same value of Γ for all materials.)

With these parameter values the dimensionless bending stiffness of the sheet, $\epsilon = B/(\Gamma R^2)$, varies in the interval $[2 \times 10^{-10}, 0.02]$.

B. Experimental protocol. All sheets were floated on the surface of water either as part of the fabrication process (for PS sheets) or manually (for other materials). Commercially available acrylic/polycarbonate spherical caps were then placed below the floating sheet and the liquid level gradually reduced until the sheet was deposited on the surface of the sphere. The system was then allowed to dry for at least ten minutes before the sphere was inspected to determine whether any delamination had occurred during the drying process. (In some instances, especially close to the transition, sheets delaminated in a portion even though experiments that are nearby in parameter space remained perfectly adhered. Such experiments are believed to be anomalous, caused by deposition not occurring axisymmetrically on the sphere, for example, but are nevertheless indicated by an open point in figures 2 and 4 of the main text for completeness.)

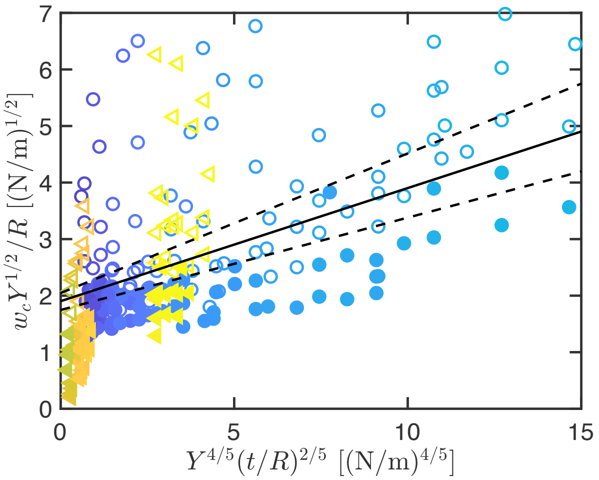

Fig. S1. Experimental data obtained with the thinnest PS and PVS sheets show that the linear relationship of Eq. (S15) is observed experimentally. The uncertainty in the value of the intercept is shown by the two dashed lines surrounding the solid line (which corresponds to $\Gamma = 0.45\ \mathrm{Nm}^{-1}$). In this figure, point colour and shape corresponds to the thickness and type of sheet, as in figure 2E,F of the main text.

C. Adhesion strength. The more detailed version of the theory presented in §1B gives more information about how the critical size for delamination is expected to behave with $\epsilon/\beta^3 \ll 1$; this information can be used in the estimation of the adhesion strength Γ, as we now discuss. In particular, we expect from Eq. (S8) that the critical sheet size for delamination with $\epsilon/\beta^3 \ll 1$ will be:

$$\frac{w_c}{R} = 2\sqrt{2}\beta^{1/2}\left[1 + c\left(\frac{\epsilon}{\beta^3}\right)^{1/5}\right] \qquad \text{[S14]}$$

with c undetermined. We note that this may be rewritten:

$$\frac{w_c}{R}Y^{1/2} = 2\sqrt{2}\Gamma^{1/2} + c'Y^{4/5}t^{2/5}R^{-2/5}\Gamma^{-3/10}. \qquad \text{[S15]}$$

We therefore expect that plotting $w_c \times Y^{1/2}/R$ versus $Y^{4/5}t^{2/5}R^{-2/5}$ will give approximately a straight line with intercept $2\sqrt{2}\Gamma^{1/2}$. Plotting the PS data in this way yields fig. S1, which shows the linear relationship expected. Fitting this linear relationship, we find that $\Gamma \approx 0.45 \pm 0.07\ \mathrm{Nm}^{-1}$ for PS (with the constant $c \approx 0.08$) and so we use the value $\Gamma \approx 0.45\ \mathrm{Nm}^{-1}$ in all of the plots presented in the main text.

D. Blister shape. The theory presented throughout the main text is based on the observation that extremely bendable sheets should form folds (rather than rucks) at the onset of the delamination instability. Profiles of delamination blisters around the periphery of the sheet (all located close to the edge of the sheet with $r/W = 0.95$) were measured using a custom made 3D scanning platform that combines a confocal distance sensor (Microepsilon IF2405) with precision linear stages (Physik Instrument), giving (in principle) horizontal and vertical resolutions of 0.5 μm and 1 μm, respectively. We also used a profilometer (Keyence: VK-X1000), reconstructing the profile from the interference pattern observed with light of wavelength 440 nm.

While the quantities of interest are the blister width and height (λ and A respectively) at an experimental level, it is difficult to measure λ directly. Instead we replace λ with the blister's full-width at half maximum, $\ell_{1/2}$. Noting that $\Delta/W \sim W^2/R^2$, we then find that in the ruck regime, we should expect:

$$\frac{A}{\lambda} \sim \left(\frac{\beta}{\epsilon^2}\right)^{1/14}\left(\frac{W}{R}\right)^{5/7}. \qquad \text{[S16]}$$

Experimental estimates of the typical aspect ratio of the blister, $A/\ell_{1/2}$, measured at the edge of the sheet are plotted in fig. 4B of the main text. These results are consistent with the prediction for the ruck regime Eq. (S16). (Here, experiments

were performed with a range of values of ϵ, β and $\tilde{\Delta} \sim W^2/R^2$. Most experiments were performed with $W/(R\beta^{1/2}) = 12 \pm 1$, corresponding to a horizontal line in the regime diagram of fig. 4A of the main text. However, for the thickest sheets, this fixed value of $W/(R\beta^{1/2})$ would not lead to delamination; experiments were therefore performed with sufficiently large $W/(R\beta^{1/2})$ that well-developed delamination occurred.)

Unfortunately, the interference techniques used did not allow for the measurement of blister profiles in the profiles for experiments with the most bendable sheets (those with the smallest values of ϵ/β^3). Nevertheless, the results shown in fig. 4 of the main text are overall consistent with the prediction that more bendable sheets should lead to delamination blisters with larger aspect ratio.

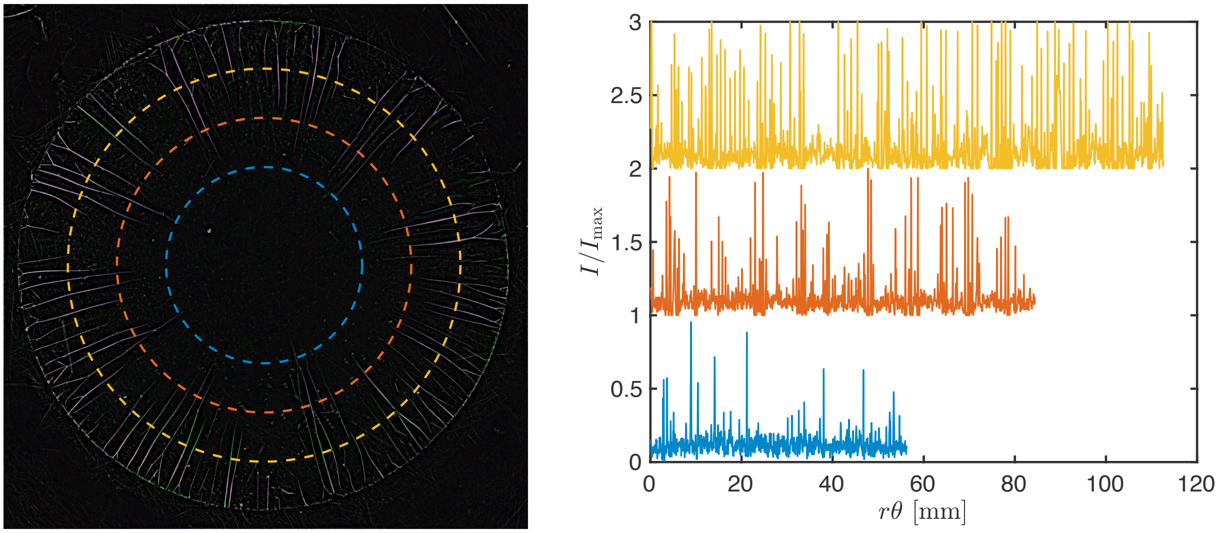

Fig. S2. Image detection of blisters at three different radial positions for a PS sheet with $\beta = 2.5 \times 10^{-4}$, $\epsilon = 8.4 \times 10^{-9}$ and $W/R = 0.21$. Left: circles are drawn at various radii (indicated by dashed curves) and the image intensity mapped along this curve. Right: The image intensity along each curve (plotted as a function of arc length) shows a series of peaks; these peaks are counted to give the number of blisters at a given radial position. (Note that the plots in the right-hand figure have been offset vertically for clarity.)

E. Spatial distribution of blisters. For regimes of parameter space in which delamination occurred, a second series of experiments was conducted in which the spatial distribution of the delamination blisters was measured. To do this, images were captured with different focal planes, and a composite image created. (An example of such a composite image is shown in fig. 1D of the main text.) From these images, the spatial distribution of the number of blisters could be determined by counting peaks in the image intensity in circles of increasing radius — such intensity peaks correspond to blisters. This process was automated via image processing techniques developed in Matlab: typically, peaks are detected when the peak intensity is more than two standard deviations from the mean of the intensity signal. For some (noisy) images, this threshold was modified to give results that accord with manual counting of peaks. An example of the composite image and the resulting intensity plots is shown in fig. S2.

References

1. B Davidovitch, RD Schroll, D Vella, M Adda-Bedia, E Cerda, A prototypical model for tensional wrinkling in thin sheets. *Proc. Natl Acad. Sci* **108**, 18227 (2011).
2. D Kumar, TP Russell, B Davidovitch, N Menon, Stresses in thin sheets at fluid interfaces. *Nat. Mater.* **19**, 690–693 (2020).
3. J Huang, et al., Capillary wrinkling of floating thin polymer films. *Science* **317**, 650–653 (2007).
